# Supplementary material for: End Sequence Analysis Toolkit (ESAT) expands the extractable information from single-cell RNA-seq data
Source: Genome Res. 2016 Oct;26(10):1397–410. doi: 10.1101/gr.207902.116 (PMC5052061; doi:10.1101/gr.207902.116)
Supplement: Supplemental Material [file supp_gr.207902.116_Supp_Materials.tar.gz › ESAT-esat_v0.1/doc/html/dir_0cb260f31f742d93669d9d6741ca4a65.html]

ESAT: java/broad/pda/samtools Directory Reference


ESAT


- Main Page
- Related Pages
- Packages
- Classes
- Files

All Classes Namespaces Files Functions Variables Enumerator Pages

- java
- broad
- pda
- samtools

samtools Directory Reference

Directory dependency graph for samtools:

|  |  |
| --- | --- |
| Files | |
| file | FilterFastqReads.java |
|  | |
| file | FilterPairedReadsByMAQ.java |
|  | |
| file | PairedEndReadIterator.java |
|  | |
| file | PairedEndTDF.java |
|  | |
| file | SAMFragmentWriter.java |
|  | |
| file | SAMPairedEndFileReader.java |
|  | |
| file | TrimFastqReads.java |
|  | |
| file | WrappedIterator.java |
|  | |


---

Generated on Fri Aug 15 2014 15:11:16 for ESAT by  

 1.8.7
